# Patient activation among patients with type 2 diabetes and its association with perception of care, perceived autonomy support, sociodemographic and clinical characteristics

**DOI:** 10.1371/journal.pone.0348840

**Published:** 2026-05-11

**Authors:** Muzlifa Mohamed Yusoff, Syahnaz Mohd Hashim, Norfazilah Ahmad

**Affiliations:** 1 Department of Family Medicine, Faculty of Medicine, Universiti Kebangsaan Malaysia, Kuala Lumpur, Malaysia; 2 Kepala Batas Health Clinic, Ministry of Health, Penang, Malaysia; 3 Department of Public Health Medicine, Faculty of Medicine, Universiti, Kebangsaan Malaysia, Kuala Lumpur, Malaysia; 4 Hospital Canselor Tuanku Muhriz, Universiti Kebangsaan Malaysia, Kuala Lumpur, Malaysia; National Healthcare Group, SINGAPORE

## Abstract

**Background:**

Patient activation is an important aspect of self-management in type 2 diabetes (T2DM) and a key component of the Chronic Care Model, whereby patients should play an active role in their care. Past studies have yet to prove the exact factors influencing patient activation. Limited studies have examined patients’ perceptions of care and whether healthcare providers are autonomy-supportive. This study primarily focused on determining factors associated with patient activation, including *sociodemographic and clinical characteristics, perception of care, and perceived autonomy support*.

**Methods:**

A cross-sectional study was conducted among T2DM patients at the public primary care clinic, Kepala Batas Health Clinic, Penang, the northern part of Malaysia, between 2^nd^ December 2024 and 30^th^ April 2025. Participants were sampled through a systematic sampling method and were given a self-administered questionnaire, comprising sociodemographic and clinical characteristics, Patient Activation Measure, Patient Assessment of Chronic Illness Care and Health Care Climate Questionnaire.

**Results:**

A total of 450 patients participated, with a response rate of 85.3%. Many were Malay (92.9%), had lower income (99.0%), attained a secondary level of education (62.0%) and were on oral hypoglycaemic agents only (OHA) (66.0%). The mean patient activation score was 59.54 (SD 14.58), and 66.7% were at a high level of activation. Multiple linear regression revealed that factors significantly associated with patient activation were male (β = 1.984, [95% CI 0.629, 3.339], *p* = 0.004), age (β = −0.089 [95% CI: −0.161, −0.017], *p* = 0.015), HbA1c (β = −6.661 [95% CI: −7.022, −6.300), *p* < 0.001] and on OHA only (β = −1.460 [95% CI: −2.902, −0.019), *p* = 0.047].

**Conclusions:**

Patients tend to have lower activation when they are older, have higher HbA1c, and are on oral hypoglycaemic agents only. However, male patients exhibit higher activation. Interventions should focus on providing targeted, tailored support to those at risk of lower activation, to enhance their engagement in diabetes self-management and improve health outcomes.

## Introduction

Type 2 Diabetes Mellitus (T2DM) is among the top ten major causes of morbidities and mortalities all over the world [1]. The rising number of T2DM cases globally has substantially strained the healthcare system [2,3]. In 2020, 445 million cases were reported and it is likely to increase to 730 million by 2050 [4]. The prevalence of diabetes in South Asia has increased from 11.29% in 2000–2004 to 22.30% in 2020–2024 [5], and in Southeast Asia, it is expected that 10.8% of adults, or one in ten, will have diabetes by 2050 [6]. Malaysia is following a similar trend, with 11.2% of adults affected in 2011 and 15.6% in 2023 [7]. Among Malaysian states, Penang has a significant number of adults with T2DM, with 106,402 individuals affected [7]. Despite extensive efforts by healthcare providers, only 34.38% of patients achieve good glycaemic control [8].

For many decades, there has been a call for a more efficient healthcare system to improve outcomes for chronic conditions, such as diabetes. Various models of care have been proposed and the most prominent is the CCM [9,10]. CCM identifies the vital components of a healthcare system that help to improve the outcomes of chronic illnesses [9,10], including Patient Activation, Goal setting, Problem-solving/Contextual Counselling, Delivery System Design/Decision Support, and Follow-up/Coordination [9,10]. A crucial component of CCM is patient activation, in which patients act as effective health managers, and to fulfil this role, they need knowledge, skills, and confidence [11,12]. The ability of patients to become health managers, i.e., ‘patient activation’ is assessed through a scale, the Patient Activation Measure (PAM) [11]. The total score of this scale can be computed and further categorised into four activation levels (levels 1-4) [11]. According to Hibbard et al. (2004), levels 1 and 2 are considered low activation, as individuals in these levels have inadequate knowledge and confidence to manage their illness. When patients reach level 3, they begin to self-manage, and at level 4, they can maintain self-management. Levels 3 and 4 are considered high activation, meaning patients are competent to become their own health managers [11]. A body of evidence showed that when patients are highly activated, their clinical outcomes, including glycaemic, lipid, and blood pressure control, improve [13–15]. This can enhance patients’ quality of life, reduce hospitalisation and healthcare costs [15].

Past research across various populations found that more than half of patients with T2DM are at a high level of activation, with a mean patient score of 54–58 [16–20]. Similar research has not yet been conducted in the local setting, as the concept of patient activation has not been widely investigated in Malaysia. Nevertheless, a local study among patients with metabolic syndrome found that the mean patient activation score was 58.9, and most were at a high activation level [21]. Previous studies have also highlighted that factors associated with patient activation include younger age [22,23], higher education level [24,25], adequate literacy skills [26,27], employment [21,24,28], and less financial distress [24,29,30]. Apart from these, having good knowledge [24,31], positive coping [32,33], depression [34], and good glycaemic control [15,25] are also significant factors. However, the outcomes of the quantitative studies showed that Nagelkerke *R*², a measure of variance (16% to 27%), was not large [17,34], suggesting that other factors remain unexplored.

It is possible that factors contributing to patient activation may not be solely based on patients’ characteristics but also on the care they receive and the attitudes of healthcare providers [35]. This view has long been expressed through the Chronic Care Model (CCM), which emphasises that patient-centred care should be established at every level of primary care, with an effective delivery system, well-organised care, and a focus on self-management and decision support [9,10]. The CCM was then examined through research, and the evidence is sufficient to demonstrate that high-quality care and positive attitudes among healthcare providers influence patients’ engagement and ability to manage their health [28,36]. In assessing patient’s perception of care, the Patient Assessment of Chronic Illness Care (PACIC) scale has been widely utilised [37,38], including for diabetes care [39].

Besides the quality of care, promoting patients’ autonomy has been shown to motivate them to engage in diabetes care [36,40,41]. Perceived autonomy support is vital in healthcare, as it promotes adherence to diabetes self-management [41-43]. This concurs with Self-Determination Theory, which posits that perceived autonomy is a fundamental element that motivates people to act [44]. The theory explains that people can thrive when they believe those in positions of authority, such as healthcare providers, allow them to make decisions about matters that are important to them, i.e., when those in authority are autonomy-supportive [44]. The concept of perceived autonomy is measured using the Health Care Climate Questionnaire (HCCQ) [40].

To date, there appears to be limited research examining the perception of care and perceived autonomy support as factors contributing to patient activation. Furthermore, efforts to assess patient activation among patients with T2DM in the local setting remain limited. Therefore, the general objective of this study was to determine the factors associated with patient activation among patients with T2DM, including *sociodemographic* and *clinical characteristics, perceptions of care,* and *perceived autonomy support*. Our secondary objective was to assess the proportions of patients with high and low levels of activation, and the mean scores for perception of care and perceived autonomy support. As Malaysia is currently experiencing a surge in T2DM cases, we hope this research will contribute to efforts to identify the most effective strategies to facilitate patient activation in diabetes self-management. Before developing an intervention, information is needed to identify potential factors, particularly from patients’ perspectives on their care and healthcare providers’ attitudes. This information is crucial, as high-quality, patient-centred diabetes care can only be delivered by a well-trained, efficient healthcare team.

## Methods

### Study design, setting and population

This cross-sectional study was conducted at Kepala Batas Health Clinic, a public primary care clinic, in the northern state of Peninsular Malaysia, Penang, from 2^nd^ December 2024–30^th^ April 2025. The inclusion criteria for this study were (i) patients with T2DM aged 18–70 years, (ii) able to read and write in Malay or English, (iii) diagnosed with T2DM for more than a year [36]. The criteria for having T2DM for more than a year were consistent with the finding that patient activation improved at one-year follow-up, when patients had sufficient time to engage with their healthcare providers [36]. The exclusion criteria were participants who had (i) an emergency condition during the visit to the clinic, (ii) cognitive or visual impairment, (iii) psychiatric illness, and (iv) physical impairment.

The sample size was calculated based on each study’s objectives. The largest sample size obtained was 357, based on the single mean formula using Z-score of 1.96 with 95% confidence interval, a margin of error of 5% [45], and a standard deviation of 0.54 for the perception of care from a previous study [46]. Considering a 20% non-response rate, 448 samples were required for this study.

### Data collection

Approval to conduct the study was obtained from the Medical and Research Ethics Committee, Faculty of Medicine UKM, Universiti Kebangsaan Malaysia (FF-2024–330) and the Ministry of Health Malaysia (NMRR ID-24–01860-YRL (IIR). Permission to perform the study was also obtained from the Penang State Health Department, the Seberang Perai Utara District Health Office, and the Family Medicine Specialist in charge.

A systematic sampling method was employed to recruit the participants. Patients with T2DM registered at the clinic’s Non-Communicable Disease Unit were identified and numbered. The principal researcher would approach every second patient and screen them for eligibility criteria. When the participants fulfilled the criteria, they were invited to participate in the study. Participants were given an information sheet, and, once they agreed to participate, they were required to sign a written consent form. Confidentiality was maintained by not including the participant’s identity. All data was entered into a password-protected computer, with the access data document restricted to the primary investigator. A self-administered questionnaire was then distributed.

### Study instrument and variables

The study used a self-administered questionnaire in both Malay and English, consisting of **five** sections: A to E. **Section A** assessed sociodemographic characteristics (*age, gender, ethnicity, marital status, educational level, employment status,* and *monthly household income*). **Section B** measured clinical characteristics *(duration of T2DM, current treatment, presence of comorbidities, number of visits to doctors per year for diabetes treatment, and recent HbA1c)*. Of note, the HbA1c result was obtained from the medical records.

**Section C** measured patients’ perceptions of care received over the past six months using the short version of the PACIC scale [38]. The brief version of this questionnaire consists of 11 items, whereby the patients were required to rate each item on an 11-point scale: *none (score of 0%)* to *always (score of 100%)*. The overall score ranged from 0 to 1100, with higher scores reflecting a better perception of care received over the past six months. The PACIC scoring method was based on the original author’s work [38]. Each participant’s average score should be obtained by adding all responses and dividing by 11, since there are 11 items in the PACIC scale. The total average score is then computed to yield the PACIC mean score [38].

**Section D** measured the extent to which doctors have been autonomy-supportive over the last six months using the short version of the HCCQ scale [47]. The brief version of this scale consists of six items, with options on a seven-point Likert Scale: *1 (not at all true)* to *7 (very true).* The overall score ranged from 6 to 42, with higher scores reflecting a greater autonomy support over the past six months. According to the original author, each participant’s average score should be calculated by summing all responses and dividing by six, since the HCCQ has six items [47]. The total average score is then computed to yield the mean HCCQ score [47,48].

**Section E** measured patient activation using the PAM scale. The scale consists of 13 items, with options on a 4-point Likert Scale: *1 (strongly disagree)* to *4 (strongly agree)* [49]. The overall scores were calculated using a 0-to-100-point algorithm scale [50]. Levels 1 and 2 signified a low level of activation, whereas Levels 3 and 4 signified a high level of activation. The validated PAM-13 questionnaire is available in English [50] and Malay [51]. Permission had been obtained to use both versions.

The **independent** variables of this study are i) sociodemographic characteristics, ii) clinical characteristics, iii) mean score of perception of care measured by the PACIC scale and iv) mean score of perceived autonomy support measured by the HCCQ scale. The **dependent** variable is the patient activation score using the PAM scale.

### Translation and validation of PACIC and HCCQ scales

The PACIC and HCCQ scales were originally developed in English and need to be translated into Malay. The translation and validation process begins with a panel of experts (*a family medicine specialist and a public health medicine specialist*) reviewing the original scale to assess its content for the local context. Next, two linguistic experts translated the English version into Malay, producing two translated Malay versions, which were then harmonised. The harmonised Malay version was subsequently translated into English by two other linguistic experts. The translated English version was then compared with the original one. The panel of experts reviewed each step of the forward and backward translations and agreed that the harmonised Malay version has a meaning similar to that of the original English. Permission was obtained from the original authors for the English version of the questionnaires and the translation of the Malay version.

Before the actual data collection, face validity was assessed in five patients with T2DM for both the English and Malay versions of the PACIC and HCCQ scales. All five patients provided feedback that both versions were well understood. Subsequently, a pilot study involving 60 T2DM patients was performed. Thirty patients completed the Malay version, while another 30 completed the English version of both the PACIC and HCCQ scales. Construct validity for the PACIC and HCCQ was assessed using independent t-test (*t* = −1.95, *df* = 58, *p* value = 0.056 and *t* = −1.54, *df* = 58, *p* value = 0.130), respectively. The reliability of both versions was determined using Cronbach’s alpha, a measure of internal consistency for both the PACIC and HCCQ scales. For the PACIC scale, the Cronbach’s alpha is 0.77 for Malay and 0.811 for English. For the HCCQ scale, the Cronbach’s alpha is 0.80 for Malay, and 0.87 for the English version. These results showed that both versions of the PACIC and HCCQ scales have good internal consistency.

### Statistical analysis

All data collected were analysed using the IBM SPSS software version 27. Categorical data were described as frequency (n) and percentage (%). Numerical data were described as the mean with standard deviation (SD) or the median with interquartile range (IQR). Statistical significance was set at *p* < 0.05. Simple linear regression was performed first to examine the association between patient activation and each of the independent variables *(age, gender, ethnicity, marital status, education level, employment status, monthly income, duration of diabetes, type of treatment, HbA1c, comorbidity status, number of visits, PACIC and HCCQ)*. Multiple linear regression was then performed, including all independent variables. Backward, forward, and stepwise methods were employed to obtain the best model. The model was reasonably well. There were no interactions among the independent variables. No multicollinearity was detected and variance inflation factors are below 10. Model assumptions were fulfilled.

## Results

### Sociodemographic and clinical characteristics

**Fig 1:**
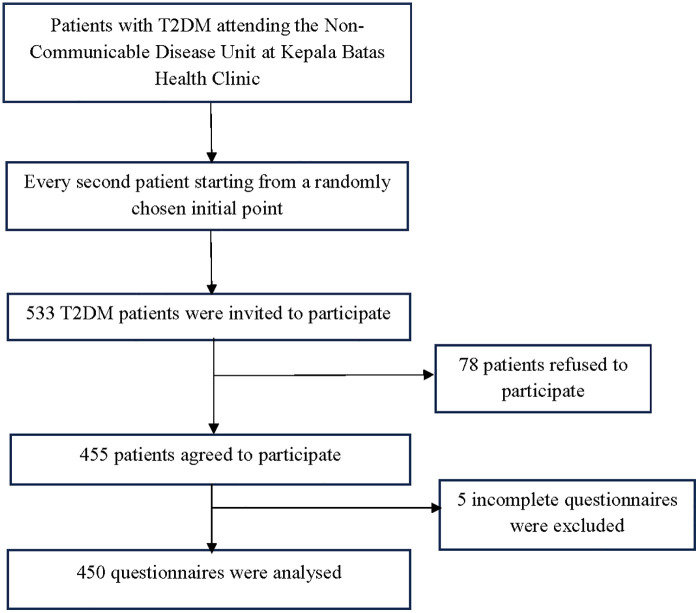
The study flow chart.

A total of 533 T2DM patients were invited to participate, of whom 455 agreed. Hence, the response rate was 85.3%. Five incomplete questionnaires needed to be excluded. Thus, only 450 questionnaires were included in the analysis. **Fig 1** details the study flow chart. **Table 1** shows that the participants’ mean age was 53.84 years (SD 9.37). The majority were Malay (92.9%), married (91.0%), and had a low monthly income (99.0%). More than half were female (57.8%), had attained up to secondary education (62.0%), and were unemployed (58.7%). Many had other comorbidities (90.0%) and 66.0% took oral hypoglycaemic agents (OHA) only. The mean duration of diabetes was 7.57 years (SD 5.53), with an average of 3.50 (SD 1.45) clinic visits for diabetes. The mean HbA1c was 8.15% (SD 1.90).

**Table 1:** Sociodemographic and clinical characteristics (N = 450).

Characteristics		n	%	Mean (SD)
**Age (years)**				53.84 (9.37)
**Age (categories)**	18-29	5	1.1	
	30-44	69	15.3	
	45-59	233	51.8	
	60-70	143	31.8	
**Gender**	Male	190	42.2	
	Female	260	57.8	
**Ethnicity**	Malay	418	92.9	
	Chinese	8	1.8	
	Indian	24	5.3	
**Marital status**	Single	15	3.3	
	Married	410	91.0	
	Divorced/Widow	25	5.6	
**Education level** ^**1**^	Primary	34	7.6	
	Secondary	280	62.0	
	Tertiary	136	30.2	
**Employment status**	Unemployed	264	58.7	
	Employed	186	41.3	
**Monthly income (MYR)** ^**2**^	Low	445	99.0	
	High	5	1.1	
**Duration of DM (years)**				7.57 (5.53)
**Type of treatment**	Lifestyle	16	3.6	
	OHA^**3**^ only	298	66.0	
	OHA^**3**^ + Insulin	136	30.2	
**HbA1c (%)**				8.15 (1.90)
**Comorbidity status** ^**4**^	No	45	10.0	
	Yes	405	90.0	
**No. of visits**				3.50 (1.45)

^1^Primary: no formal education & primary school, Secondary: secondary school, Tertiary: diploma, college, matriculation, degree, masters, PhD. ^2^Low: those with monthly income of <RM5,250, High: those with monthly income of ≧ RM5,250, Based on Department of Statistics Malaysia 2025. ^3^OHA: oral hypoglycaemic agents. ^4^Comorbidity status: having other chronic illnesses besides diabetes.

### Patient activation and level

The mean score of patient activation is 59.54 (SD 14.58). Out of 450 patients, 300 (66.7%) were at high activation level, Level 3 and 4, as demonstrated in **Table 2**.

**Table 2:** Patient Activation, Perception of Care and Perceived Autonomy Support.

Variables	n	%	Mean (SD)
Patient Activation (PAM score)			59.54 (14.58)
Low Patient Activation level (1&2)	150	33.3	
High Patient Activation level (3&4)	300	66.7	
Perception of care (PACIC score)			65.23 (14.89)
Perceived autonomy support (HCCQ score)			5.54 (0.98)

### Perception of care and perceived autonomy support

The mean scores for perception of care and perceived autonomy support were 65.23 (SD 14.89) and 5.54 (SD 0.98), respectively, as displayed in **Table 2**.

### Factors associated with patient activation

**Table 3** presents the preliminary factors significantly associated with patient activation, including male, Malay, tertiary education, duration of diabetes, use of OHA (oral hypoglycaemic agents) only, use of both OHA and insulin, HbA1c, absence of comorbidity, number of clinic visits, perception of care, and perceived autonomy support.

**Table 3:** Preliminary Factors Associated with Patient Activation.

Factors		Simple Linear Regression			
		β	95% CI	t	*p*-value
**Age (years)**		−0.006	−0.151, 0.138	−0.088	0.930
**Gender**	Female (reference)				
	Male	3.615	0.899, 6.331	2.615	0.009*
**Ethnicity** ^**1**^	Non-Malay (reference)				
	Malay	7.949	2.742, 13.157	3.000	0.003*
**Marital status** ^**2**^	Single/ Divorced/Widow (reference)				
	Married	−3.484	−8.223, 1.255	−1.445	0.149
**Education level**	Primary (reference)				
	Secondary	−1.789	−4.572, 0.995	−1.263	0.207
	Tertiary	4.633	1.721, 7.545	3.127	0.002*
**Employment status**	Unemployed (reference)				
	Employed	2.141	−0.597, 4.879	1.537	0.125
**Monthly income (MYR)** ^**3**^	Low (reference)				
	High	4.931	−7.957, 17.819	0.752	0.452
**Duration of DM (years)**		−0.351	−0.593, −0.108	−2.840	0.005*
**Type of treatment**	Lifestyle modification (reference)				
	OHA^4^ only	3.268	0.426, 6.110	2.260	0.024*
	OHA + Insulin	−4.347	−7.263, −1.431	−2.930	0.004*
**HbA1c (%)**		−6.637	−6.995, −6.278	−36.389	<0.001*
**Comorbidity status**	Yes (reference)				
	No	8.011	3.567, 12.455	3.543	<0.001*
**No. of visits**		−1.300	−2.225, −0.375	−2.761	0.006*
**Mean PACIC score**		0.236	0.147, 0.324	5.249	<0.001*
**Mean HCCQ score**		2.393	1.034, 3.753	3.460	<0.001*

^1^Ethnicity: Chinese and Indian were categorised into non-Malay. ^2^Marital status: single/divorced/widow were categorised into single. ^3^Low: those with monthly income of <RM5,250, High: those with monthly income of ≧RM5,250, Based on Department of Statistics Malaysia 2025. ^4^OHA: oral hypoglycaemic agents ^*^Significant at p < 0.05. Simple Linear Regression: (Normality and equal variances assumptions for all variables were met and independent random samples were drawn for the construction of data).

**Table 4** presents the final four factors that were significantly associated with patient activation. Older patients have a lower patient activation, of 0.089 [(95% CI −0.161, −0.017), *p* = 0.015], while males exhibit a higher patient activation [β = 1.984 (95% CI 0.629, 3.339), *p* = 0.004] compared to females. Those with higher HbA1c have a lower patient activation score, of 6.661 [(95% CI −7.022, −6.300), *p* < 0.001]. Similarly, patients on OHA only had a lower patient activation [β = 1.460 (95% CI −2.902, −0.019), *p* = 0.047] compared to those on lifestyle modification. The model explains 75.9% of the variance of the mean patient activation in patients with T2DM (adjusted *R*^2^ = 0.759).

**Table 4:** Final Factors Associated with Patient Activation among T2DM Patients.

Factors		Multiple Linear Regression		
	β	95% CI	t	*p*-value
Age (years)	−0.089	−0.161, −0.017	−2.442	0.015*
Male (reference: female)	1.984	0.629, 3.339	2.877	0.004*
Taking OHA only (reference: lifestyle modification only)	−1.460	−2.902, −0.019	−1.991	0.047*
HbA1c (%)	−6.661	−7.022, −6.300	−36.235	<0.001*

^4^OHA: oral hypoglycaemic All predictor variables are included for variable selection in the multiple linear regression. ^*^Significant at *p* < 0.05 Backward Multiple Linear Regression was applied. The model fits reasonably well. Model assumptions were fulfilled. There was no interaction among the independent variables. No multicollinearity was detected. Adjusted *R*^2^ = 0.759.

## Discussion

### Patient activation score and level

The current study is among the initial local efforts to assess patient activation among patients with T2DM and provides insight into factors contributing to it at a primary care setting. Our results demonstrated a mean patient activation score of 59.54, consistent with another local study reporting a score of 59.4 [21]. The figure is also close to other Asian studies in Singapore (58.8) [26] and Saudi Arabia (55.9) [25]. However, findings from the United States and Finland have shown that the mean patient activation score is higher, 63.2 [14] and 69.9 [25], respectively. Although our mean patient activation score is not remarkably high, more than half of the participants (66%) are at a high activation level, corresponding to Levels 3 and 4. The result aligns with prior research [21,25,26], indicating that patients with T2DM strive to acquire a high level of knowledge, skills, and confidence to self-manage. This is significant, as highly activated patients would know their needs, likely be engaged, and be empowered to self-manage [11,12,18]. Consequently, the healthcare system would become more patient-centred, higher-quality and cost-effective. Patient activation has been recognised as a cornerstone of modern chronic care models, empowering individuals to move from passive recipients of medical services to active partners in the healthcare system [11,12].

### Factors associated with patient activation

In this study, four factors were significantly associated with patient activation: ***age, male gender, taking OHA only, and HbA1c***. These four factors account for 75.9% of the variance in patient activation, a figure higher than that reported in other research [17,34]. The final analysis revealed that as age increases, the patient activation score decreases. Our findings appear to replicate those of previous studies, suggesting that elderly patients may be less able to manage their diabetes [26,29]. They may have multiple health issues that can affect their physical capacity and motivation to engage in diabetes self-care [52–54]. Furthermore, older patients may have inadequate financial resources, as some choose to retire after reaching 65 or 70 years of age [55,56]. As a result, they may lack the capacity to serve as effective health managers and are unable to handle any unexpected problems related to diabetes. This is concerning, as in our local setting, elderly patients often lack sufficient financial resources to purchase a glucometer for blood sugar monitoring or to attend frequent visits to check for the development of target organ damage [2,57]. All these aspects can influence their activation in diabetes self-management.

The current study also demonstrated that males exhibit better patient activation than females. The finding is consistent with studies reporting that men are more confident and more likely to be engaged in managing their diabetes [58,59]. They appear to be adopting a “proactive health manager” approach, actively monitoring blood glucose and integrating self-care into their daily routines [60,61]. This behaviour may result from their determination to remain healthy, as they need to fulfil their role as the breadwinner.

Apart from the above factors, we found that patients on oral medications had lower activation than those on lifestyle modification. The result is similar to previous research that linked patient activation to treatment regimens [25,62]. Perhaps the complexity of the treatment regimens contributed to the patient’s limited understanding, confidence, and ability to self-manage diabetes [63–66]. Past research has shown that patients may not adhere to medication regimens when the dose and schedule are too complex [63,65].

Based on our analysis, glycaemic control, as measured by HbA1c, emerged as a significant factor associated with patient activation. It has been demonstrated that higher HbA1c levels are associated with lower patient activation scores, consistent with other studies [25,67]. Patients with poor glycaemic control are likely to be less adherent to self-care tasks, such as dietary recommendations and blood glucose monitoring [25]. Meanwhile, those with better control have higher activation, as they are more likely to take an active role and feel confident and knowledgeable about managing their diabetes. Nevertheless, the relationship between HbA1c and patient activation may be bidirectional, with patient activation influencing HbA1c. This opinion is based on a published work stating that a high level of patient activation is associated with better HbA1c [67].

The results highlighted that most T2DM patients have a moderate level of perception of care, with a mean PACIC score of 65.23 (SD 14.89). The finding aligns with other local surveys, indicating that the care delivered to patients with T2DM has not yet achieved its optimal level [46,68]. More effort is needed to ensure that our local patients receive well-structured diabetes care, with an effective delivery system and treatment plan. This fact has been emphasised by many scholars from other parts of the world [69–72]. The current study is among the initial efforts to investigate the relationship between patient activation and perception of care among patients with T2DM. In contrast to past studies [28,46], our study found no link between patient activation and patient perception of care, as measured by the PACIC scale. Perhaps this contrasting result is due to the different scales used: the current study utilised the shorter PACIC scale (11 items), whereas the studies employing the 20-item scale [28,46].

In the current study, participants perceived their healthcare providers as autonomy-supportive, as indicated by a high overall mean HCCQ score, consistent with other research [43,73,74]. This finding is important to us, as it reveals that patients viewed healthcare providers as respectful, listened to their opinions, and tried to understand their views and choices. These aspects are crucial for building trust and empowering patients to manage diabetes [43,73,74]. Although the link between perceived autonomy and diabetes self-management was evident in a study by Chen et al. (2022), our study found the opposite [43]. Perhaps having autonomy over one’s own health is not sufficient for patient activation; rather, a high level of knowledge and self-efficacy in self-management are key elements of activation.

## Strengths, limitations and research recommendations

One notable strength of this study is that it represents an early local effort to explore factors associated with patient activation among patients with T2DM, offering valuable insights into a relatively understudied area. However, several limitations should be considered when interpreting the findings. Firstly, the study was conducted at a single centre, which may limit the generalisability of the results to wider populations or diverse healthcare settings. Secondly, the cross-sectional design of the study limits the ability to draw causal conclusions about the examined variables.

Future research employing longitudinal designs would be beneficial in clarifying the temporal and potentially causal nature of these associations. To enhance the applicability and relevance of findings, future research should consider nationwide studies employing a multicentre design that includes a more diverse population across various healthcare settings.

## Conclusion and implications for clinical practice

Based on our study, most patients with T2DM exhibit a high level of activation. Factors significantly associated with patient activation include *age, male gender, taking oral hypoglycaemic agents only* and *HbA1c*. These findings highlight the significant influence of demographic and clinical characteristics on patient engagement in diabetes self-management. Certain aspects, such as age, gender, glycaemic control, and the complexity of treatment regimes, play a crucial role in determining patient activation. Therefore, it is essential to provide targeted, tailored support to groups at risk of lower activation, particularly older adults, women, individuals with poor glycaemic control, and those on complex treatment regimens. Such personalised interventions could help enhance their engagement, improve self-care practices, and ultimately lead to better health outcomes.

## Supporting information

S1 File Data in excel. (XLSX)
